# Revisiting the Model Human Processor: a neurophysiological investigation based on P300 and Bereitschaftspotential

**DOI:** 10.3389/fnhum.2025.1690746

**Published:** 2025-10-28

**Authors:** Toshitaka Higashino, Naoki Wakamiya

**Affiliations:** Graduate School of Information Science and Technology, The University of Osaka, Suita, Japan

**Keywords:** Model Human Processor, P300, Bereitschaftspotential, dual-process theory, reaction time, EEG

## Abstract

**Introduction:**

The Model Human Processor (MHP), while useful, lacks direct neurophysiological validation. This study aimed to validate and extend the MHP by analyzing P300 and Bereitschaftspotential (BP) brainwave components.

**Methods:**

Our initial finding of qualitatively different neural signatures between correct and incorrect trials led to the hypothesis that the “correct” trial group is a mixture of different processing types. We tested this by segregating correct trials based on the presence or absence of the P300 component, which we reasoned is a key marker of the MHP’s conscious “Initiate Response” process.

**Results:**

We identified a P300-absent subgroup even among correct responses. This subgroup exhibited significantly shorter reaction times than its P300-present counterpart and showed a neural signature strikingly similar to that of incorrect trials, including a delayed negative peak in the BP.

**Discussion:**

These results suggest the human information processing pathway is not monolithic. We propose a new model that bifurcates after perception into either a “Deliberate Process” (P300-present), which aligns with the MHP, or a high-speed “Automatic Process” (P300-absent) that bypasses the MHP’s “Initiate Response” process. This work provides neurophysiological validation for the MHP and lends new neural support for dual-process theory.

## 1 Introduction

Humans act by repeating a “perception-cognition-action” cycle (Neisser, 2014). To elucidate the mechanisms of this cycle, multifaceted analyses have been advanced in the field of psychology (Ganz, 1975; Harter, 1967; Fitts and Posner, 1967). The outcomes of this research have been applied to engineering fields, playing a particularly crucial role in the design of user interfaces (UI) and user experiences (UX). Designing a superior UI/UX requires decomposing and analyzing user behavior process by process to optimize elements such as button placement and response times (Norman, 1988; Rasmussen, 1983).

However, applying these findings to diverse situations requires elevating them from specific experimental contexts to generalized models. This is because the results of individual psychological studies are often reported under limited conditions, making their reproducibility and applicability in different environments challenging (Brunswik, 1956).

A representative attempt at this generalization is the Model Human Processor (MHP) proposed by Card et al. (1983). The MHP conceptualizes a human as a type of information processing system and systematizes findings from psychological experiments into a form that is applicable to engineering. In the MHP, the human information processing course is broadly divided into three processors: the Perceptual Processor, which receives external stimuli; the Cognitive Processor, which makes decisions based on the received information; and the Motor Processor, which executes actions based on cognitive judgments.

**FIGURE 1:**
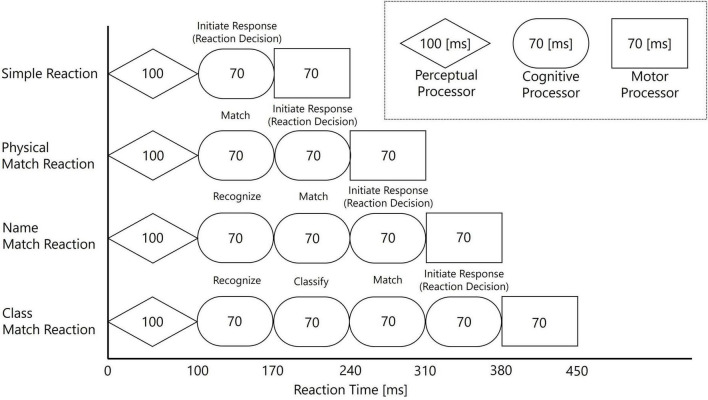
Schematic of the Model Human Processor (MHP) framework used for the task design in Experiment 2. The diagram illustrates how the four tasks, from Simple Reaction to Class Match, were designed by incrementally adding cognitive sub-processes (“Match,” “Recognize,” “Classify,” “Initiate Response”) to the basic perceptual-cognitive-motor sequence. The processing times for each stage and the overall predicted reaction times are based on the standard model proposed by Card et al. (1983).

The Cognitive Processor, responsible for complex thought and decision-making, is further broken down into multiple sub-processes within the MHP. Representative processes include matching, character recognition, classification, and “Initiate Response,” each with a standard processing time assigned. These processes are defined as follows: matching involves a simple physical comparison between a stimulus and a target (e.g., “Is this stimulus the letter B?”); character recognition requires identifying a stimulus by its name regardless of physical form (e.g., identifying both “D” and “d” as the same character); and classification entails categorizing a stimulus based on abstract rules (e.g., “Is this letter a consonant?”). To measure these processes, this study employs tasks designed to incrementally engage them, thereby varying cognitive complexity. A key feature of the MHP is its proposal of four processing models corresponding to task complexity, achieved by combining these processors and processes. A schematic of this MHP framework, and the corresponding task design for this study, is illustrated in Figure 1.

Since its proposal, the MHP has been extended in various forms. For instance, extended models have been proposed for specific engineering purposes, such as the Queuing Network-Model Human Processor (QN-MHP), which introduces queuing network theory to model situations involving parallel processing of multiple pieces of information, unlike the single-process assumption of the MHP (Liu et al., 2006). Another is MHP/RT, which more validly predicts human behavior in complex, real-world environments beyond responses to limited stimuli (Kitajima and Toyota, 2013).

However, the MHP and these extended models share a critical limitation: they are constructed by reverse-engineering from behavioral outcomes such as reaction time, and their processing stages do not directly reflect actual brain activity. The processing times assumed for each processor and process in the MHP are merely estimates derived from behavioral observation. To make the MHP a model with higher validity, it is necessary to validate its processing stages from the perspective of brain activity and to incorporate the obtained findings back into the model. While numerous studies have been conducted to validate the MHP, those approaching it from a neurophysiological standpoint remain scarce.

To address this challenge, our previous research attempted to validate the MHP’s Cognitive Processor using electroencephalography (EEG) (Higashino and Wakamiya, 2021). Our focus was on the P300 component, a positive-going event-related potential (ERP) peaking approximately 300 ms or more after a stimulus. The P300 is not a monolithic component; it is often divided into the earlier, fronto-centrally distributed P3a, associated with novelty detection, and the later, parietally-maximal P3b, linked to target categorization and context updating in memory (Barry et al., 2020; Polich, 2012). The latency of the P3b, in particular, is widely considered to reflect the duration of stimulus evaluation (Sutton et al., 1965; Polich, 2007, 2012), making it a powerful tool for indexing cognitive processing time. Its utility as a robust measure of cognitive function is further demonstrated by its use as a clinical assay and its correlation with higher-order cognitive performance (Polich and Herbst, 2000; Privitera and Sun, 2024). In our prior experiment, we confirmed that as reaction time increased in accordance with MHP models, the P3b latency also systematically delayed, leading us to conclude that it is a powerful index of the “Initiate Response” process (Higashino and Wakamiya, 2021). However, with the P300 alone, while we could capture the endpoint of the cognitive process, we could not examine the transition from cognition to motor action in detail.

To overcome this limitation, the present study introduces a functionally distinct neurophysiological marker: the Bereitschaftspotential (BP), or readiness potential. Unlike the stimulus-locked P300 which reflects cognitive evaluation, the BP is a response-locked, slow negative potential that begins up to 2 s before a voluntary movement, reflecting the planning and preparation processes within the motor cortex (Libet et al., 1983; Shibasaki and Hallett, 2006). The P300 and BP are thus powerful and complementary indices for dissecting the perception-cognition-action cycle: the P300 marks the culmination of stimulus-driven cognitive processing, while the BP marks the start of self-initiated motor preparation.

The unique, complementary nature of the P300 (marking the end of cognition) and the BP (marking the start of motor action) allows us to neurophysiologically dissect the MHP’s proposed stages. Therefore, this study was designed as a two-part investigation. First, in Experiment 1, we conduct a crucial methodological validation to confirm that our experimental setup can reliably elicit and measure the P300 and BP components in isolation. This foundational step ensures the integrity of our core measurements.

With this validated methodology, Experiment 2 then proceeds to the main investigation, which addresses our primary research questions. We examine how the neural signatures of cognitive evaluation (P300 latency) and motor preparation (BP onset) change as task complexity increases in accordance with the MHP’s predictions. Furthermore, we investigate the specific temporal relationship between the completion of cognitive processing and the onset of motor preparation, and how this relationship varies with task difficulty. Ultimately, these neurophysiological findings will be used to test the MHP’s assumption of a single, monolithic processing pathway and to question whether the data reveals evidence for alternative processing routes that would necessitate an extension to the conventional model. By addressing these questions, we aim to provide a neurophysiological validation of the MHP and, if necessary, build a new, more realistic information processing model that can account for the dynamic interplay between cognition and action.

To achieve these goals, this study will test two primary hypotheses:

Hypothesis A: Incorrect responses are generated by a faster process than correct responses, resulting in significantly shorter reaction times.

Hypothesis B: The group of correct trials is not neurally homogeneous, but is instead a mixture of an MHP-compliant “Deliberate Process” and a rapid “Automatic Process.”

## 2 Experiment 1

### 2.1 Methods

#### 2.1.1 Participants

The participants in this study were ten young adults (9 males and 1 female, mean age 23.4 years) with normal hearing and vision capabilities. This sample size was deemed appropriate for the study’s nature as an exploratory investigation aimed at generating hypotheses regarding the neurophysiological basis of the proposed model. Data collection was conducted with the approval of the Ethics Committee of the Graduate School of Information Science and Technology, The University of Osaka, Japan. All participants were informed of the details of the experiment including possible risks and participant rights in advance, and the experiments were conducted after obtaining their consent.

#### 2.1.2 Experimental setup

In all experiments, participants were seated. The distance from the participant’s head to a display showing visual stimuli was 1 m. The display was a 24-inch LCD monitor (Dell P2412Hb). All participants grasped and freely used a computer mouse with their right hands.

#### 2.1.3 Tasks and procedure

This experiment consisted of two distinct tasks designed to reliably elicit the P300 and Bereitschaftspotential (BP) components, respectively.

##### 2.1.3.1 Task 1-1 (oddball task)

To elicit the P300 component, a visual oddball paradigm was employed. Participants were presented with a random sequence of standard stimuli (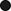 symbol) and rare target stimuli (▲ symbol). The ratio of target to standard stimuli was 1:3. Stimuli were presented at intervals varying randomly between 1000 and 2000 ms, with each stimulus being preceded by a fixation cross (+ symbol). A single block consisted of 124 total stimulus presentations. To isolate cognitive evaluation from motor execution, participants were instructed to silently count the number of target stimuli without making any physical response. After the block, they reported their count to confirm task engagement. This task was performed once by each participant.

##### 2.1.3.2 Task 1-2 (self-paced movement task)

To measure the BP, a self-paced button-press task was used. A fixation cross (+ symbol) was continuously displayed on the screen. Participants were instructed to perform button presses at their own pace, at arbitrary and self-chosen intervals, for the duration of the task. The task lasted for 180 s, although this duration was not disclosed to the participants. The precise timing of each button press was recorded. This task was also performed once by each participant.

#### 2.1.4 EEG recording and preprocessing

Electroencephalography data were recorded during all experimental tasks using an OpenBCI Cyton system equipped with ThinkPulse Active Electrodes. The sampling rate was 256 Hz. Data were acquired from the Cz and C3 electrode sites according to the international 10–20 system (Jasper, 1958). The Cz electrode was selected for P300 analysis as the P3b component is typically maximal over midline centro-parietal scalp locations (Polich, 2012). For the BP, the C3 electrode was chosen as it overlies the left motor cortex, which is contralateral to the right hand used for the button-press response, where the BP is known to be maximal (Shibasaki and Hallett, 2006). Electrodes placed on the left and right mastoids served as the reference, and an electrode on the forehead (Fp1) served as ground.

Offline analysis of the EEG data was performed using MATLAB. The raw data were first bandpass filtered between 1 and 30 Hz. The continuous data were then segmented into epochs for each trial. Artifact rejection was performed in a two-step procedure. First, trials containing significant artifacts, including ocular artifacts (e.g., eye blinks and movements), muscle activity, or other transient noise, were manually rejected via visual inspection. This manual approach was chosen to ensure careful and precise data cleaning appropriate for this exploratory dataset, rather than relying on automated component-based methods like ICA which can be less reliable with a limited number of channels. Second, a statistical outlier rejection was applied to the remaining trials. Specifically, for each electrode, any trial with an amplitude exceeding ±2 standard deviations from the mean of that trial’s baseline period was defined as an artifact and excluded from further analysis.

#### 2.1.5 EEG analysis

Electroencephalography data from Experiment 1 were analyzed separately for each task to isolate the P300 and BP components. All analyses were performed after the preprocessing steps described above.

For Task 1-1, the analysis focused on the P300 component elicited by target stimuli. Data from the Cz channel were segmented into epochs ranging from -200 ms to 800 ms, time-locked to the onset of the target stimulus. These epochs were then baseline-corrected using the pre-stimulus interval (-200 ms to 0 ms) and averaged for each participant to create an individual ERP waveform.

For Task 1-2, the analysis targeted the BP preceding self-initiated button presses. Data from the C3 channel were epoched from −1500 to 500 ms, time-locked to the onset of the button press. These epochs were then averaged for each participant to visualize the BP waveform.

### 2.2 Results

The analysis confirmed that the experimental paradigms in Experiment 1 successfully elicited the P300 and BP components.

**FIGURE 2:**
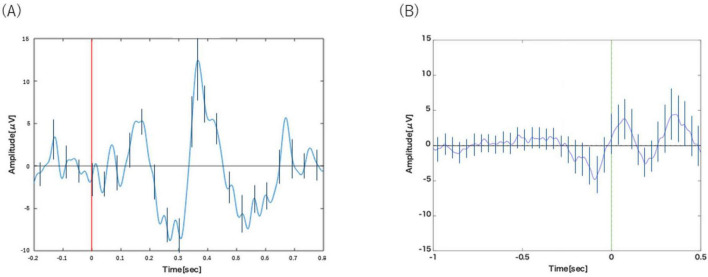
**(A)** Analysis of Task 1-1. P300 was observed around 400 ms after the visual stimulus. **(B)** Analysis of Task 1-2. BP was observed from about 1000 to 200 ms before the motion. Error bars represent the standard deviation.

Figure 2A displays the grand-averaged ERP waveform from the Cz channel in Task 1-1. The waveform represents the average of a mean of 28.9 ± 0.57 trials (Mean ± SE) per participant. Following the presentation of target stimuli, a prominent positive-going peak, characteristic of the P300 component, was observed at a latency of approximately 400 ms.

Figure 2B displays the grand-averaged waveform from the C3 channel in Task 1-2, time-locked to the button press. The waveform represents the average of a mean of 20.2 ± 1.23 trials (Mean ± SE) per participant. A slow negative-going potential, characteristic of the Bereitschaftspotential (BP), was clearly visible preceding the motor response, beginning at approximately −1000 ms.

### 2.3 Brief discussion

The primary objective of Experiment 1 was to verify that our experimental setup and analytical pipeline could reliably elicit and accurately measure the two distinct neurophysiological components central to this study: the P300 and the Bereitschaftspotential (BP).

The results confirm the successful validation of our methodology. In Task 1-1, the classic oddball paradigm elicited a prominent positive peak around 400 ms post-stimulus (Figure 2A), which is morphologically and temporally consistent with the P300 component. This demonstrates our ability to isolate the cognitive processes associated with stimulus evaluation and decision-making, independent of motor execution. In parallel, Task 1-2 successfully captured a slow, negative-going potential shift preceding self-initiated movements (Figure 2B), a hallmark signature of the BP that reflects motor preparation.

By demonstrating that our methods can effectively capture both the cognitive-evaluative P300 and the motor-preparatory BP in isolation, we established a robust methodological foundation. This validation was a critical prerequisite, ensuring that the findings in the main experiment would be attributable to the dynamic interplay between these processes rather than to measurement artifact. We could therefore proceed with confidence to Experiment 2, where these functionally distinct components were measured concurrently to investigate their relationship within the Model Human Processor framework under varying task demands.

## 3 Experiment 2

### 3.1 Methods

#### 3.1.1 Participants

The same ten young adults who participated in Experiment 1 also took part in Experiment 2.

#### 3.1.2 Experimental setup

The experimental setup, including the display, viewing distance, and response device, was identical to that described in Experiment 1.

#### 3.1.3 Tasks and procedure

Experiment 2 comprised four tasks designed to progressively engage additional cognitive subprocesses within the Model Human Processor (MHP) framework. With the exception of the Simple Reaction Task, these tasks were based on the oddball paradigm–which requires a response to infrequent target stimuli–to observe how neurophysiological markers change with increasing cognitive complexity. The tasks ranged from the Simple Reaction Task (Task 2-1) to the more complex Class Match Reaction Task (Task 2-4), with each subsequent task intended to add a specific layer of cognitive processing. In all tasks, visual stimuli were presented at random intervals between 1000 and 2000 ms, preceded by a fixation cross. The order of the four tasks was randomized for each participant.

##### 3.1.3.1 Task 2-1: Simple Reaction Task

This task was designed to measure the most basic MHP pathway, involving only the “Initiate Response” process within the Cognitive Processor. Participants were presented with a target stimulus (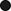 symbol) 30 times. They were instructed to press a button with their right hand as quickly as possible upon seeing the stimulus.

##### 3.1.3.2 Task 2-2: Physical Match Reaction Task

This task added a “Match” process to the MHP pathway. A series of letters (from the set A, B, C, a, b, c, 1, 2, 3) was presented one at a time. Participants were instructed to press the button only when the specific target letter “B” appeared and to do nothing for other stimuli. The task consisted of 120 trials, with a target-to-non-target stimulus ratio of 1:3.

##### 3.1.3.3 Task 2-3: Name Match Reaction Task

Building on the previous task, this one incorporated a “Recognize” process, requiring participants to identify stimuli by name rather than by physical form. Letters were presented from a larger set (A, B, C, D, E, a, b, c, d, e, 1, 2, 3, 4, 5). Participants were instructed to press the button whenever a letter pronounced “/di:/” was shown, meaning they had to respond to both “D” and “d.” The task included 120 trials with a 1:3 target-to-non-target ratio.

##### 3.1.3.4 Task 2-4: Class Match Reaction Task

This was the most complex task, designed to engage all cognitive subprocesses in the model, including “Classify.” Using the same stimulus set as Task 2-3, participants were instructed to press the button whenever a consonant letter (B, C, D, b, c, or d) was presented. This required them to classify each letter before responding. The task consisted of 120 trials with a 1:3 target-to-non-target ratio.

#### 3.1.4 EEG recording and preprocessing

Electroencephalography recording and preprocessing procedures were identical to those described in Experiment 1.

#### 3.1.5 Data analysis plan

The data analysis in this study aimed to clarify the effects of task complexity and response accuracy on behavioral performance and neurophysiological indices (P300 and BP). Both EEG data processing and statistical analysis were conducted using custom scripts in MATLAB. The statistical significance level was set at *p* < 0.05 for all tests.

##### 3.1.5.1 Trial categorization

In each task of Experiment 2 (Task 2-2, Task 2-3, and Task 2-4), trials were classified into three categories based on the presented stimulus and the participant’s response. Category X (Correct Hits) included trials in which a target stimulus was presented and the participant correctly made a button-press response. Category Y (Misses) consisted of trials in which a target stimulus was presented, but the participant failed to make a button-press response. Finally, Category Z (False Alarms) comprised trials in which a non-target stimulus was presented, yet the participant incorrectly made a button-press response. Note that Task 2-1 (Simple Reaction Task) included only Category X trials, as no non-target stimuli were presented.

##### 3.1.5.2 Behavioral data analysis

Reaction time (RT) was analyzed as the primary behavioral measure. Mean RTs for Category X and Category Z were calculated for each participant in each task. To test Hypothesis A, mean RTs for Category X and Category Z were compared within each task (Task 2-2, Task 2-3, and Task 2-4) using paired-samples *t*-tests.

##### 3.1.5.3 EEG data processing and analysis

The primary objective of the neurophysiological analysis was the concurrent measurement of the P300 and BP components. To achieve this, the same trial data was epoched and analyzed in two different ways using different time-locking events. For the P300 analysis, data from the Cz channel were epoched from −200 to 800 ms, time-locked to the stimulus onset (0 ms), and the component was quantified by its peak latency (the maximum positive amplitude) within a 250–600 ms post-stimulus window. For the BP analysis, data from the C3 channel were epoched from −1500 to 500 ms, time-locked to the button-press response (0 ms), and the component was characterized by the onset latency of the slow negative potential and its negative peak latency. For visualization purposes in figures superimposing the two waveforms, the response-locked BP waveform was time-shifted backward by the mean RT for that condition, converting it to a stimulus-locked timeline for direct comparison.

##### 3.1.5.4 Hypothesis-driven re-analysis of correct trials

The initial observation that the neural activity of correct and incorrect trials was qualitatively different motivated a direct test of Hypothesis B. To test this hypothesis, we re-analyzed Category X trials by classifying them based on the presence or absence of the P300 component. The subgroup classification was performed for each trial; if a positive peak exceeding +2 standard deviations from the baseline mean was detected at the Cz channel within the 250–600 ms post-stimulus window, the trial was classified as “P300-present” (X + P3). Trials that did not meet this criterion were classified as “P300-absent” (X−P3). Following this classification, a comparative analysis was performed. Average waveforms were created for the X + P3 and X−P3 subgroups, and paired-samples *t*-tests were conducted in each task to compare their reaction times.

### 3.2 Results

The behavioral and electroencephalography (EEG) data were analyzed according to the procedures outlined in the Data Analysis Plan. The number of artifact-free trials included in the analysis for each condition is summarized in Table 1. This table confirms that a sufficient number of trials were secured for the analyses; critically, the participant contributing the fewest trials still provided at least seven trials for the key “Automatic Process” (X−P3) and six trials for the false alarm (Z) conditions.

**TABLE 1:** Descriptive statistics for the number of artifact-free trials per condition.

Task	Correct (X)	Correct (X + P3)	Correct (X−P3)	False alarm (Z)
Task 2-1 (Simple)	28.4 (1.2) Range: (26–30)	–	–	–
Task 2-2 (Physical)	23.4 (1.9) Range: (19–28)	18.9 (1.6) Range: (16–22)	4.5 (1.0) Range: (3–6)	3.2 (1.2) Range: (1–5)
Task 2-3 (Name)	20.5 (2.4) Range: (15–26)	15.3 (2.0) Range: (12–19)	5.2 (1.3) Range: (3–7)	6.0 (1.6) Range: (4–9)
Task 2-4 (Class)	17.8 (2.7) Range: (11–24)	9.5 (2.2) Range: (5–13)	8.3 (1.6) Range: (6–11)	8.7 (1.8) Range: (6–12)

Descriptive statistics for the number of artifact-free trials per condition across all participants (*N* = 10). Each cell shows the mean, standard deviation in parentheses, and the range (minimum–maximum).

As is evident from the table, the number of correct trials (X) systematically decreased as task difficulty increased. Notably, in the most difficult Class task, the proportion of correct trials attributed to the “Automatic Process” (X−P3) increased substantially, becoming nearly equal to the number of trials from the “Deliberate Process” (X + P3).

#### 3.2.1 Behavioral results

**FIGURE 3:**
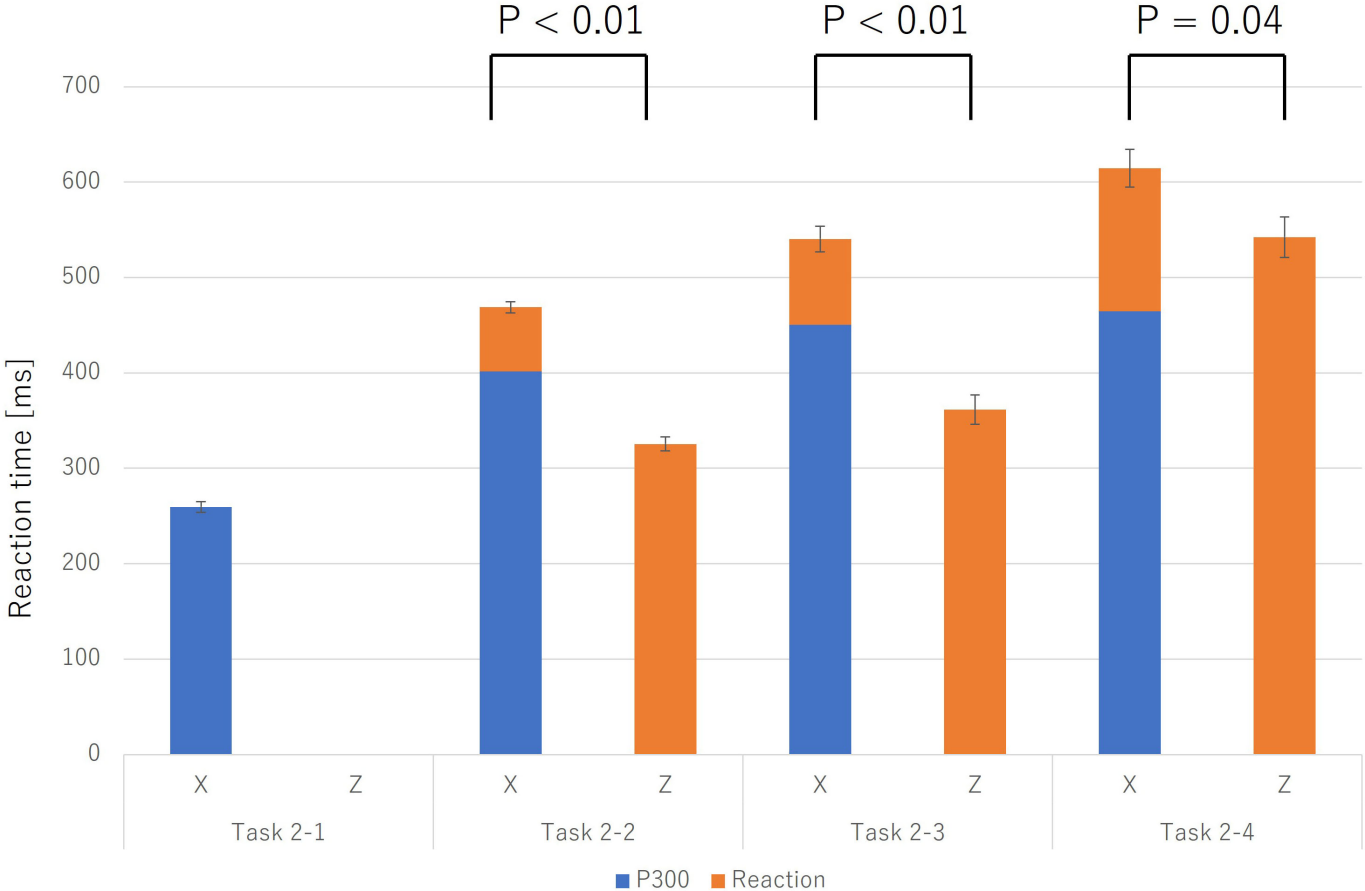
Reaction times for incorrect trials (Z; solid orange bars) were significantly shorter than for correct trials (X; stacked bars) across Task 2-2, Task 2-3, and Task 2-4 (*p* < 0.05). For correct trials (X), the stacked bar is broken down into the P300 latency (the blue portion), which reflects the completion of the “Initiate Response” process, and the subsequent time until the response (the orange portion). Error bars represent the standard error. This finding suggests that incorrect trials may be triggered by more impulsive processes that bypass deliberative judgment.

Reaction times (RTs) for correct trials (Category X) systematically increased with task complexity. A more critical finding emerged when comparing RTs between correct (Category X) and incorrect trials (Category Z). As shown in Figure 3, RTs for incorrect trials were consistently shorter than for correct trials.

To statistically validate this, paired-samples *t*-tests were conducted for each task. The results confirmed that the mean RT for incorrect trials was significantly shorter than for correct trials in the Physical Match Reaction Task (Task 2-2) (Mean ± SE: *X* = 468.82 ± 5.92 ms, *Z* = 325.60 ± 7.24 ms; t(9) = 14.52, *p* < 0.001, Cohen’s *d* = 4.59), the Name Match Reaction Task (Task 2-3) (*X* = 540.43 ± 13.36 ms, *Z* = 368.45 ± 15.39 ms; t(9) = 11.01, *p* < 0.001, Cohen’s *d* = 3.48), and the Class Match Reaction Task (Task 2-4) (*X* = 614.42 ± 19.88 ms, *Z* = 553.38 ± 21.24 ms; t(9) = 2.31, *p* = 0.046, Cohen’s *d* = 0.73). These findings, particularly the very large effect sizes observed across all tasks, strongly suggest that incorrect responses were not the result of prolonged deliberation but rather of a more rapid, impulsive process.

#### 3.2.2 EEG results: correct trials (Category X)

In correct trials (Category X), both the P300 and Bereitschaftspotential (BP) components were clearly observed across all four tasks. The analysis revealed a systematic effect of task complexity on the timing of these components.

**FIGURE 4:**
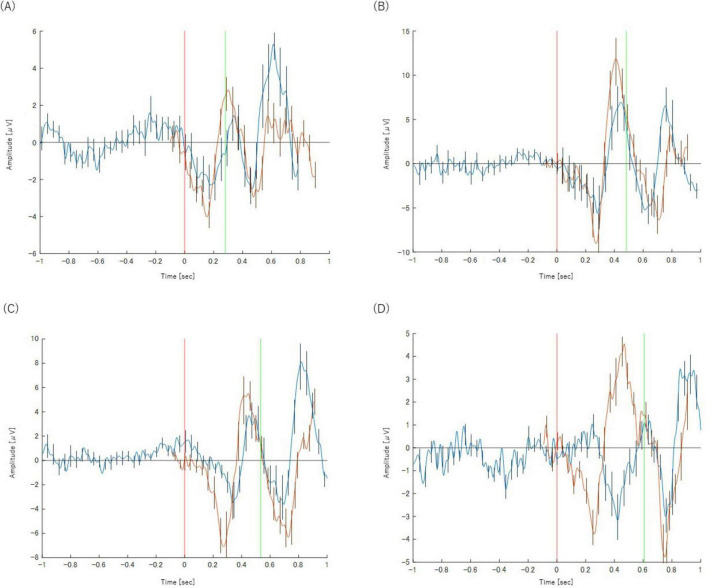
Panels show the results for **(A)** Task 2-1 (X), **(B)** Task 2-2 (X), **(C)** Task 2-3 (X), and **(D)** Task 2-4(X), respectively. The orange waveform represents the P300 analysis and the blue waveform represents the BP analysis. Both are plotted on a common time axis relative to stimulus presentation (*t* = 0, indicated by the red vertical line). The time axis for the BP waveform has been adjusted for comparison, as described in the text. The green vertical line indicates the mean reaction time for each task. Error bars represent the standard deviation.

The peak latency of the P300 component showed a progressive delay as the task became more complex, increasing from approximately 300 ms in the Simple Reaction Task (Task 2-1) to 500 ms in the Class Match Reaction Task (Task 2-4). A similar pattern was observed for the BP. The onset of the motor preparation potential was also systematically delayed with increasing task difficulty, shifting from approximately −200 ms relative to the stimulus in Task 2-1 to 250 ms post-stimulus in Task 2-4. These neurophysiological changes corresponding to task complexity are illustrated in Figure 4. The key latency metrics for each task are summarized in Table 2.

**TABLE 2:** Key metrics for P300 and BP in each task (Category X).

Task	P300	BP	
	Latency (ms)	Onset latency (ms)	Peak latency (ms)
Task 2-1	300	−200	100
Task 2-2	400	−180	200
Task 2-3	450	−50	200
Task 2-4	500	250	200

#### 3.2.3 EEG results: incorrect trials (Categories Y and Z)

In stark contrast to correct trials, the neural activity for incorrect trials (both Category Y and Z) was qualitatively different. The EEG patterns for both types of errors–misses (Y) and false alarms (Z)–were remarkably similar to each other.

**FIGURE 5:**
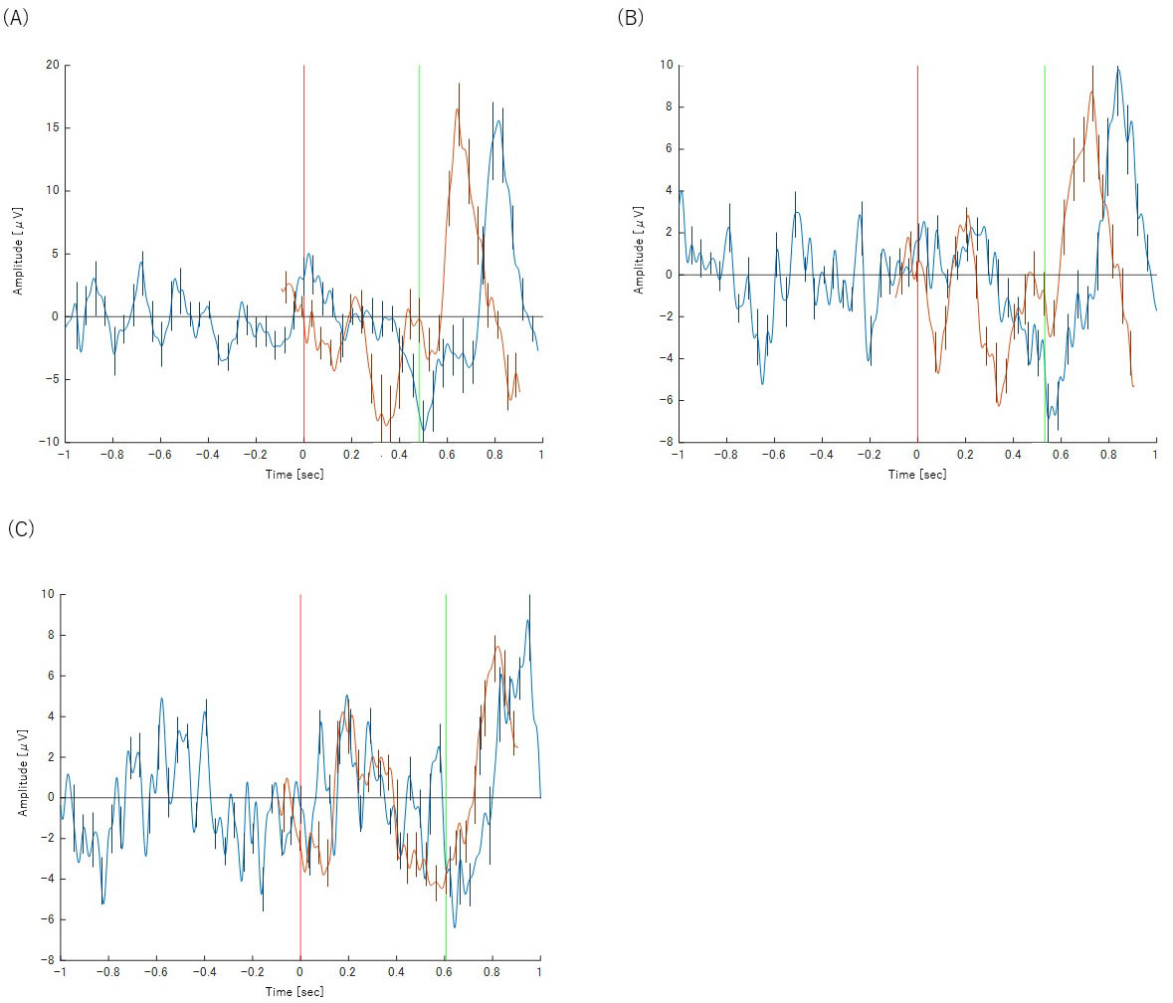
Panels show the results for **(A)** Task 2-2 (Y), **(B)** Task 2-3 (Y) and **(C)** Task 2-4 (Y), respectively. The orange waveform represents the P300 analysis and the blue waveform represents the BP analysis. Both are plotted on a common time axis relative to stimulus presentation (*t* = 0, indicated by the red vertical line). The time axis for the BP waveform has been adjusted for comparison, as described in the text. The green vertical line indicates the mean reaction time for each task. Error bars represent the standard deviation.

**FIGURE 6:**
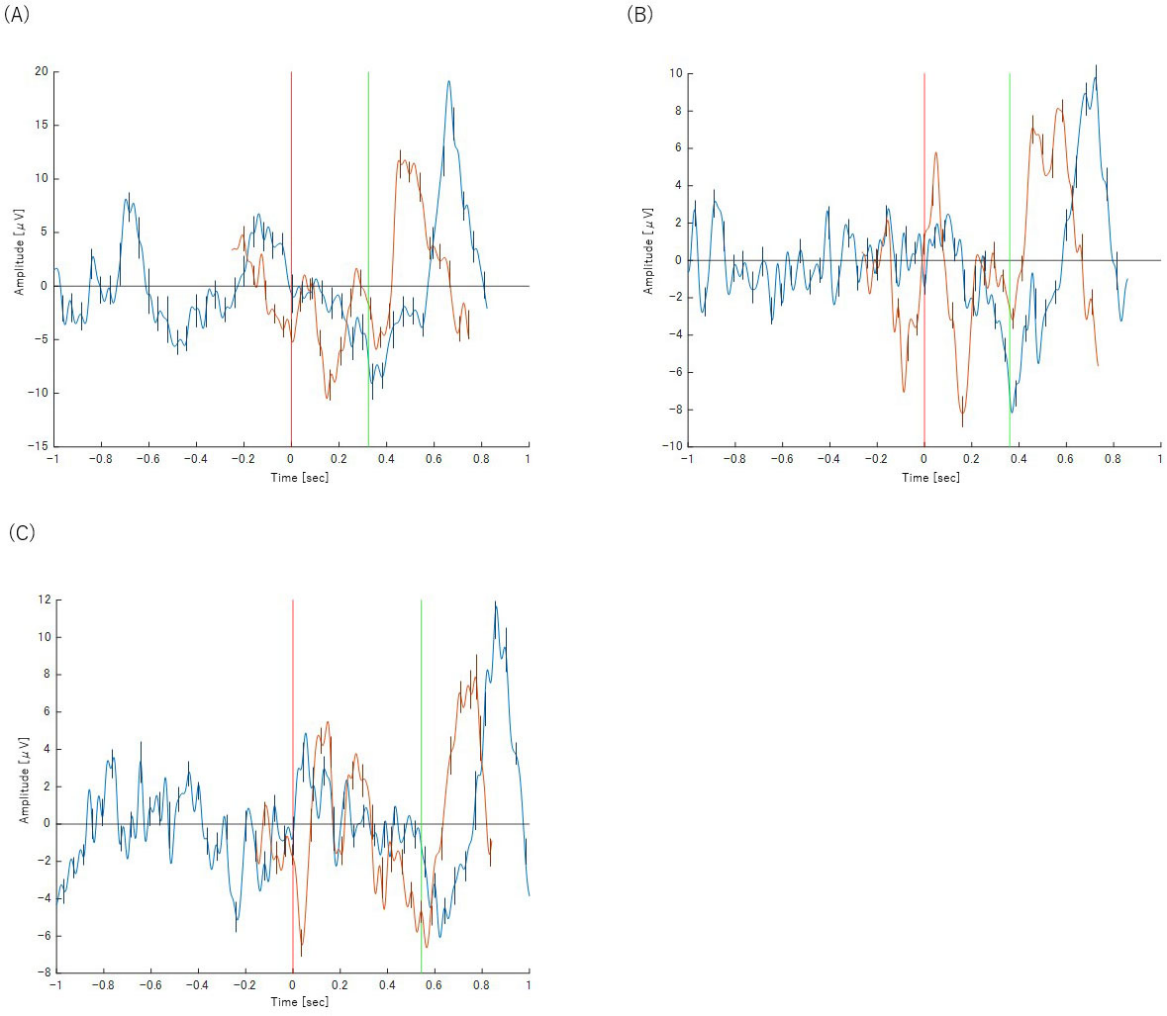
Panels show the results for **(A)** Task 2-2 (Z), **(B)** Task 2-3 (Z) and **(C)** Task 2-4 (Z), respectively. The orange waveform represents the P300 analysis and the blue waveform represents the BP analysis. Both are plotted on a common time axis relative to stimulus presentation (*t* = 0, indicated by the red vertical line). The time axis for the BP waveform has been adjusted for comparison, as described in the text. The green vertical line indicates the mean reaction time for each task. Error bars represent the standard deviation.

The most significant difference was the complete absence of a discernible P300 component in any of the incorrect trial categories. While a Bereitschaftspotential (BP) was observed, its morphology was notably different from that of correct trials. Instead of a slow, preparatory potential, the negative peak of the BP occurred much later, almost simultaneously with the button-press response itself. This suggests a lack of the deliberate motor preparation seen in correct trials. The waveforms for these trials are presented in Figures 5, 6.

#### 3.2 4 Re-analysis of correct trials based on P300 presence

To directly test Hypothesis B, correct trials were re-analyzed after being segregated into two subgroups based on the presence (X + P3) or absence (X−P3) of a P300 component. This analysis revealed a P300-absent subgroup (X−P3) whose neural and behavioral profile was strikingly different from the P300-present (X + P3) group.

**FIGURE 7:**
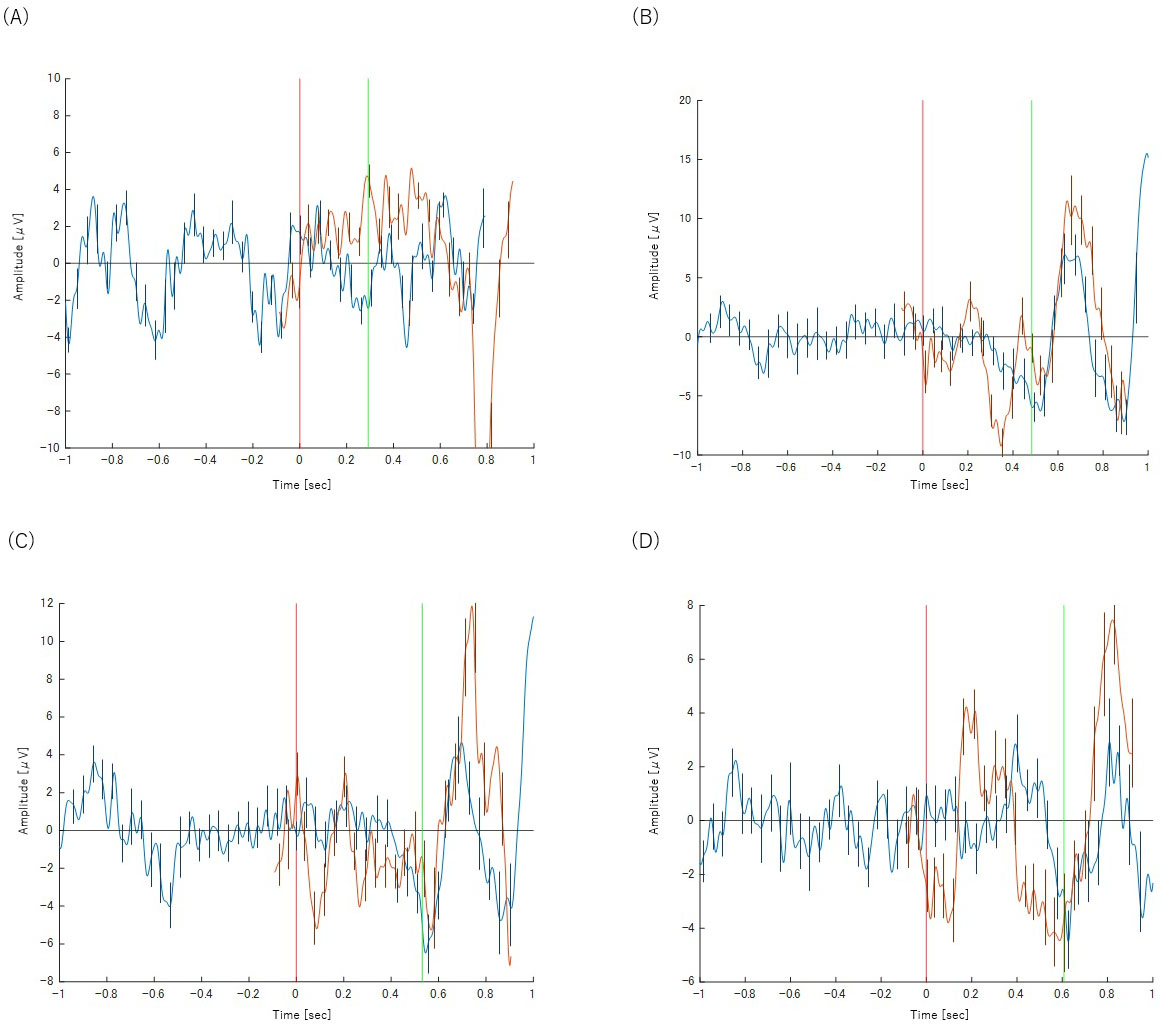
Panels show the results for **(A)** Task 2-1 (X–P3), **(B)** Task 2-2 (X–P3), **(C)** Task 2-3 (X–P3), and **(D)** Task 2-4 (X–P3) respectively. The orange waveform represents the P300 analysis and the blue waveform represents the BP analysis. Both are plotted on a common time axis relative to stimulus presentation (*t* = 0, indicated by the red vertical line). The time axis for the BP waveform has been adjusted for comparison, as described in the text. The green vertical line indicates the mean reaction time for each task. Error bars represent the standard deviation.

Neurally, the X−P3 subgroup was remarkably similar to incorrect trials (Category Z). As depicted in Figure 7, these P300-absent trials showed a BP waveform with a late negative peak occurring almost simultaneously with the motor response, a pattern inconsistent with deliberate preparation.

**FIGURE 8:**
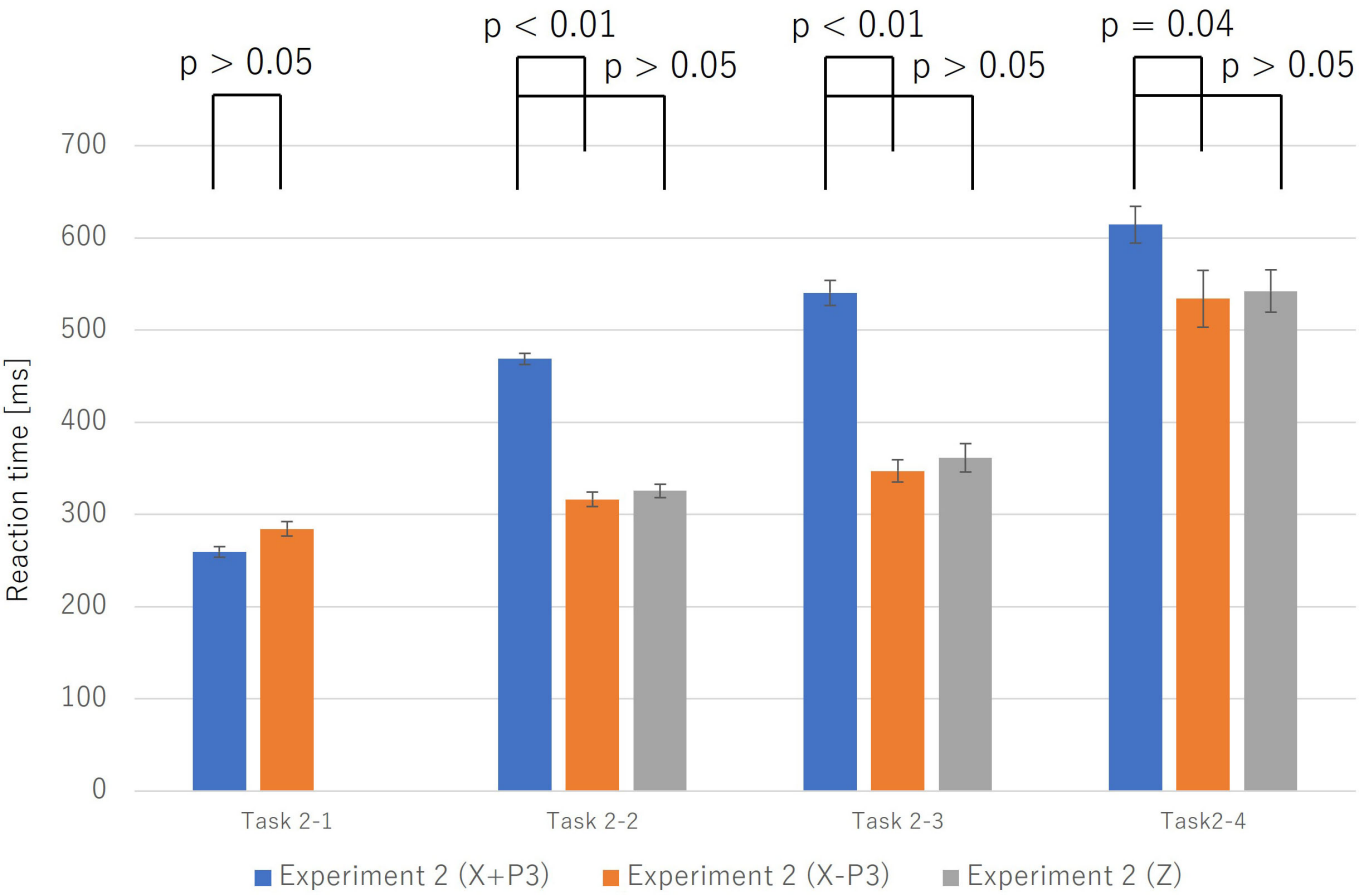
This graph compares reaction times among three groups: correct trials with a P300 component (X + P3; blue), correct trials without a P300 (X–P3; orange), and incorrect trials (Z; gray). Error bars represent the standard error. In Task 2-2, Task 2-3, and Task 2-4, reaction times for the X–P3 group were significantly shorter than for the X + P3 group. Furthermore, there was no significant difference in reaction time between the X–P3 and Z groups. This suggests that correct responses without a P300 follow the “Automatic Process” similar to that of incorrect responses.

This neural similarity was mirrored in the behavioral data. A comparative analysis of RTs (Figure 8) revealed that the X−P3 group was significantly faster than the X + P3 group across all tasks (e.g., Name Match: t(9) = 9.51, *p* < 0.001, Cohen’s *d* = 3.01). Crucially, there was no significant difference between the reaction times of the fast-correct X−P3 group (Mean ± SE: 354.00 ± 12.23 ms) and the incorrect Z group (368.45 ± 15.39 ms), t(9) = −0.94, *p* = 0.371, Cohen’s *d* = −0.30.

These results strongly suggest that a subset of correct responses is achieved via a rapid, “Automatic” pathway that is neurophysiologically and behaviorally indistinguishable from the one that produces incorrect responses.

## 4 Discussion

The principal finding of this study is the neurophysiological identification of two distinct processing pathways–a deliberate, P300-present route and a rapid, P300-absent automatic route–coexisting even within correctly executed trials. This discovery challenges the notion of a single, monolithic information processing stream and provides a more nuanced model of human cognition.

Our results demonstrate that the similarity between “automatic correct” trials (X−P3) and “incorrect” trials (Z) is not coincidental. Both are characterized by the absence of the P300 component and a late-peaking BP, and their reaction times are statistically indistinguishable. This strongly suggests that both outcomes arise from a common underlying mechanism: a high-speed, automatic pathway that bypasses the conscious “Initiate Response” process indexed by the P300. This pathway can be viewed as a high-risk, high-rewards strategy; when the automatic impulse is correct, it results in a rapid, efficient success (X−P3), but when it is wrong, it results in an impulsive error (Z).

These findings have significant implications for the Model Human Processor (MHP). While the traditional MHP effectively describes the deliberate, P300-present pathway, its assumption of a single processing stream fails to account for the automatic pathway observed in our data. We therefore propose an extension to the MHP: a “bifurcation model” where a decision point exists after the initial perceptual process. Evidence for a common pathway prior to this bifurcation is supported by the observation of the N100 component across all trials and by prior neuroimaging studies showing no significant differences in brain activity immediately preceding the P300 regardless of its eventual appearance (Rusiniak et al., 2013; Clark et al., 2000).

**FIGURE 9:**
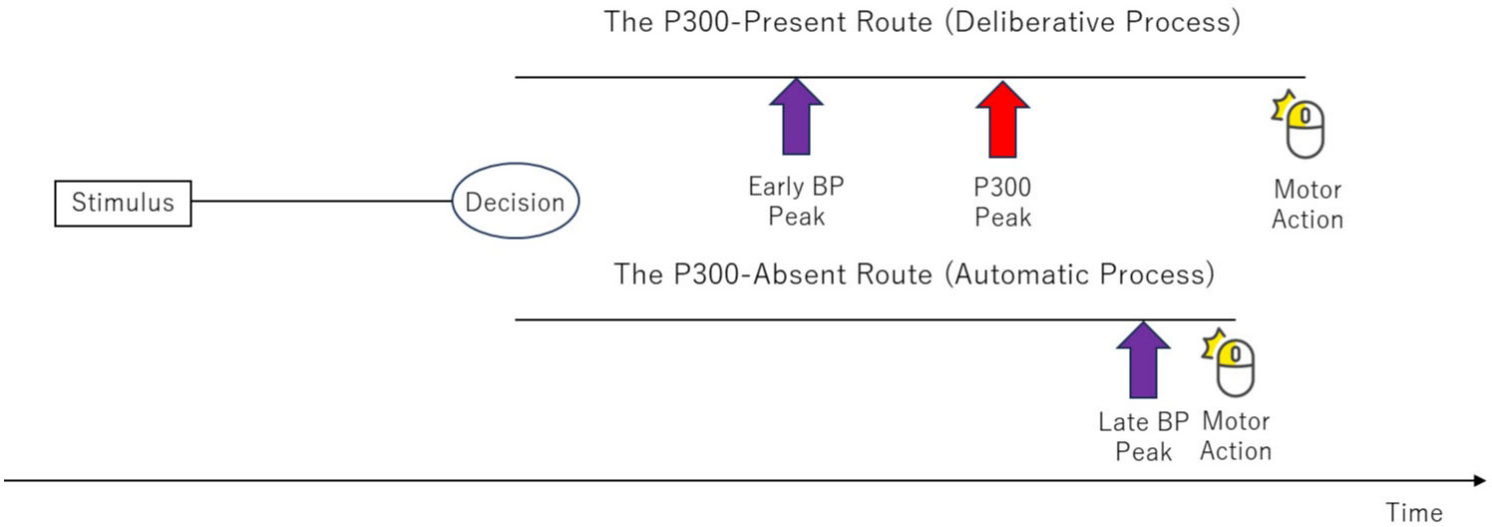
Schematic timeline of neurophysiological events following the bifurcation point. In the “Deliberate Process” (upper path), the BP peak is observed before the P300 peak. This temporal order demonstrates that the decision to select a pathway must occur before the onset of motor preparation (indicated by the BP). In the “Automatic Process” (lower path), the BP peak occurs much later, almost simultaneously with the motor action, and the P300 is absent.

The temporal sequence of neural events helps to locate this bifurcation point. In the deliberate route, the BP peak precedes the P300 peak, which implies that the decision to select a pathway is made before the conscious “Initiate Response” process is complete (Figure 9).

**FIGURE 10:**
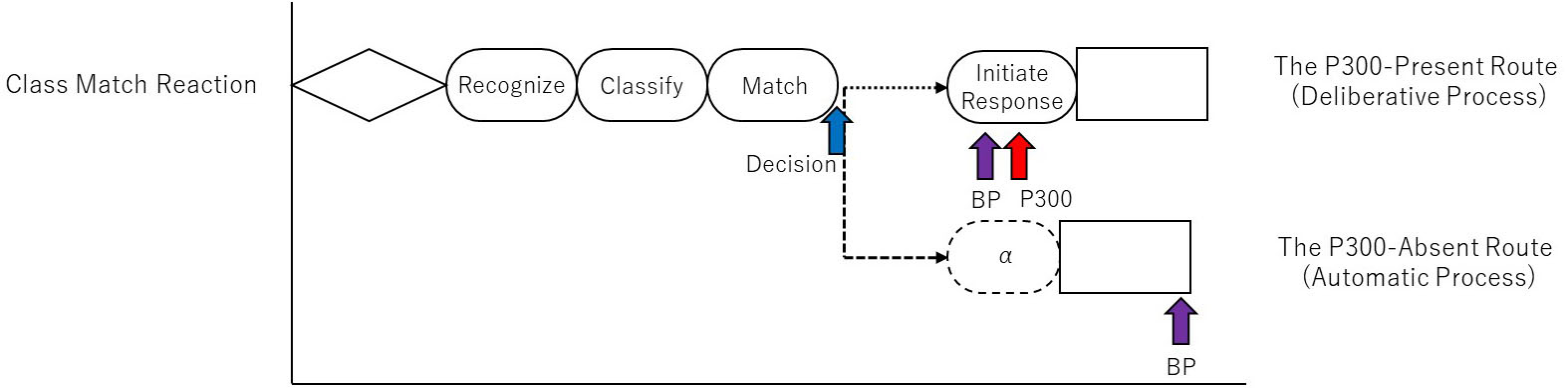
A schematic of the new information processing model that extends the MHP, based on the findings of this study. The diagram uses the most complex task, the Class Match Reaction, as an example to illustrate the processing pathways. The model posits a bifurcation point after the perceptual process, where a decision is made on “whether to execute the “Initiate Response” process or not.” The upper pathway represents the “Deliberate Process”, which includes the “Initiate Response” process, corresponds to the traditional MHP, and is indexed by the presence of the P300. The lower pathway represents a high-speed “Automatic Process” that skips this stage, resulting in the absence of the P300 and a shorter reaction time. The dotted arrows indicate the connection between processes, and the arrows themselves do not represent time.

At this bifurcation point, the system either engages the deliberate, MHP-compliant route or diverts to the high-speed, automatic route, as schematized in Figure 10. These two routes are as follows:

(1)    The P300-present route (“Deliberate Process”): This pathway aligns with the traditional MHP. It involves a preparatory (early) BP, followed by the completion of the “Initiate Response” process (P300 peak), leading to a considered motor action.
(2)    The P300-absent route (“Automatic Process”): This pathway bypasses the “Initiate Response” stage. The result is a shortened reaction time and a late-peaking BP that occurs almost simultaneously with the motor action.

It is important, however, to interpret the link between the late-peaking BP and our proposed “Automatic Process” with caution. While this pattern is consistent with our dual-process framework, alternative explanations for a late BP peak exist. For example, this neural signature could reflect a heightened sense of motor urgency or response conflict preceding an impulsive action, rather than a qualitatively different processing route. The present evidence is correlational, and our study was not designed to differentiate between these potential mechanisms. Therefore, while our findings provide novel neurophysiological evidence for a dual-pathway model, we acknowledge that attributing the late BP definitively to an “Automatic Process” is an interpretation that requires further investigation.

Furthermore, this proposed dual-pathway structure resonates strongly with the broader framework of Dual-Process Theory. The deliberate, P300-present pathway corresponds well with the slow, effortful operations of “System 2,” while the automatic, P300-absent pathway mirrors the fast, intuitive nature of “System 1” (Kahneman, 2011; Stanovich and West, 2000). Our study contributes to this field by providing concrete neurophysiological markers that may distinguish these two modes of thinking. The absence of the P300 in the automatic route aligns with modern models of it as a “build-to-threshold” decision variable (Twomey et al., 2015); the automatic pathway appears to bypass this evidence accumulation entirely. Similarly, the late BP peak provides novel support for modern interpretations of motor initiation as a stochastic process, where the decision to move occurs much closer to the action itself (Schurger et al., 2012).

### 4.1 Limitations and future directions

While this study provides neurophysiological evidence for a bifurcated model of human information processing, it is important to acknowledge several limitations that also point toward promising avenues for future research.

First, the study was conducted with a small and gender-imbalanced sample of only ten participants (9 males, 1 female). This composition limits the generalizability of our findings, and the results may not fully represent the broader population. While we reported effect sizes to provide a measure of the magnitude of our results, the statistical power to detect smaller effects was limited. Therefore, a primary direction for future work is to replicate these findings with a larger, more gender-balanced, and diverse cohort to ensure the robustness and generalizability of the proposed model.

Second, while our model proposes a bifurcation into deliberate and automatic pathways, the factors that determine the selection of either route on a given trial remain unknown. Future experiments should therefore systematically manipulate variables such as cognitive load, time pressure, or task familiarity to elucidate the dynamic mechanisms that govern the switch between these two processing modes.

Finally, the “Automatic Process” in our model is currently defined only by the absence of the P300 component at a single electrode (Cz), which is a simplified approach. We acknowledge that interpreting neurophysiological phenomena from a single channel has limitations and that unsupervised, multi-electrode approaches may provide a more comprehensive characterization of underlying cortical processes (Ghosh et al., 2024). Furthermore, the specific neural mechanisms that operate in place of the MHP’s “Initiate Response” process remain unelucidated. A critical future objective is to employ a multi-modal approach, combining EEG with other neuroimaging methods like fMRI or MEG, to clarify the neural basis and computational role of this high-speed processing route. Addressing these limitations will lead to a more comprehensive understanding of the flexible and dynamic nature of human information processing.

## 5 Conclusion

The objective of this study was to validate the Model Human Processor (MHP), a model of human information processing, using the P300 and BP brainwave components as indices, and subsequently, to construct a new, extended model. While we initially proceeded to test a hypothesis regarding a “re-evaluation process” following the “Initiate Response,” our investigation ultimately revealed more fundamental, dynamic aspects of human information processing.

The principal findings of this study can be summarized in the following three points.

First, we demonstrated that trials resulting in correct responses and those ending in incorrect responses do not merely represent the success or failure within a single process. Instead, they follow qualitatively different information processing pathways, as typified by the presence or absence of the P300. Incorrect trials are likely the result of more impulsive “Automatic Process” that lacks a careful “Initiate Response” process.

Second, as our most significant discovery, we revealed that even within correctly performed trials, a mixture of two distinct information processing pathways coexists. One is the “Deliberate Process” that aligns with the MHP and undergoes the “Initiate Response” process (indexed by the P300). The other is the “Automatic Process” that shortens reaction time by skipping this decision stage. This discovery was made possible by our study’s unique analytical approach of segregating correct trials based on the presence or absence of the P300.

Third, by integrating these findings, we propose a new “bifurcation model” that extends the conventional MHP. This model posits that a bifurcation point exists after the perceptual process, where a decision is made on “whether to execute the “Initiate Response” process or not.” This choice determines the subsequent processing pathway (deliberate or automatic) and the corresponding neural activity (presence or absence of P300, timing of BP) and behavior (reaction time).

The significance of this study lies in its demonstration, based on neurophysiological indices, that human thought and decision-making do not always follow a single, rational process. Instead, they constitute a flexible system that dynamically utilizes two modes deliberation and automatic depending on the situation. This not only contributes to the refinement of the MHP model but also provides new empirical evidence for the dual-process theory in cognitive science. Furthermore, the bifurcation into a deliberate and an automatic route that we propose may reflect the dynamic engagement of the cognitive control network (e.g., medial frontal gyrus, anterior cingulate cortex), which a recent meta-analysis of fMRI studies has shown to be consistently activated during dual-process tasks (Gronchi et al., 2024).

However, limitations remain in this study. In particular, the specific processing content and neural basis of the “Automatic Process” (P300-absent route) in our proposed model remain unelucidated. Clarifying the conditions under which this high-speed, efficient pathway is selected and the mechanisms by which it is executed is an important topic for future research.

## Data Availability

The datasets presented in this article are not readily available because the informed consent did not include the declaration regarding publication of data. Requests to access the datasets should be directed to toshitaka-higashino@ist.osaka-u.ac.jp.
